# Health profiles of foreign-born elderly women with HIV in Italy

**DOI:** 10.3389/fragi.2025.1659751

**Published:** 2025-09-29

**Authors:** Stefania Arsuffi, Eugenia Quiros-Roldan, Fabio Riccardo Colombo, Benedetta Fioretti, Caterina Candela, Benedetto Maurizio Celesia, Micol Ferrara, Jovana Milic, Giuseppe Vittorio De Socio, Giordano Madeddu, Anna Maria Cattelan, Stefania Piconi, Paolo Bonfanti, Agostino Riva, Giovanni Guaraldi, Stefano Calza, Andrea Calcagno, Emanuele Focà, GEPPO Cohort, Arsuffi Stefania, Azzolino Domenico, Baroni Marta, Bellelli Giuseppe, Bonaffini Luca, Bonfanti Paolo, Calcagno Andrea, Calza Stefano, Cattelan Annamaria, Celesia Benedetto Maurizio, Coin Alessandra, De Socio Giuseppe, Di Perri Giovanni, Ferrara Micol, Fioretti Benedetta, Focà Emanuele, Guaraldi Giovanni, Italiani Francesca, Lazzaro Alessandro, Lucchi Tiziano, Grazia Maddalone Maria, Madeddu Giordano, Marengoni Alessandra, Mastroianni Claudio, Milić Jovana, Mussi Chiara, Nozza Silvia, Orofino Giancarlo, Patetta Lavinia, Piazzoli Andrea, Piconi Stefania, Pignata Paola, Pontiggia Silvia, Riva Agostino, Spolti Anna

**Affiliations:** 1 Department of Clinical and Experimental Sciences, Unit of Infectious Diseases, University of Brescia and Spedali Civili Hospital, Brescia, Italy; 2 Unit of Biostatistics and Bioinformatics, Department of Molecular and Translational Medicine, University of Brescia, Brescia, Italy; 3 Clinic of Infectious Diseases, San Raffaele Scientific Institute, Milano, Italy; 4 Division of Infectious Diseases, ARNAS Garibaldi, Catania, Italy; 5 Unit of Infectious Diseases, Department of Medical Sciences, University of Torino, Torino, Italy; 6 Department of Surgical, Medical, Dental and Morphological Sciences, University of Modena and Reggio Emilia, Modena, Italy; 7 Department of Medicine, Clinic of Infectious Diseases, University of Perugia, Perugia, Italy; 8 Unit of Infectious Diseases, Department of Medicine, Surgery and Pharmacy, University of Sassari, Sassari, Italy; 9 Infectious and Tropical Diseases Unit, Padua University Hospital, Padova, Italy; 10 Infectious Diseases Unit, Alessandro Manzoni Hospital, Lecco, Italy; 11 Fondazione IRCCS San Gerardo dei Tintori, Monza - University of Milano-Bicocca, Milan, Italy; 12 Third Division of Infectious Diseases, University of Milan, Ospedale L. Sacco, Milan and University of Milan, Milan, Italy

**Keywords:** HIV, women, foreign-born, aging, healthy longevity

## Abstract

**Background:**

Ageing trajectories for foreign-born individuals and women living with HIV remain poorly defined globally. This study aimed to characterize foreign-born women living with HIV aged ≥65 years (FWLH) and compare them to age-matched Italian women (IWLH) and foreign-born men living with HIV (FMLH).

**Methods:**

Data were drawn from the multicenter Italian geriatric HIV cohort (GEPPO). We described sociodemographic characteristics, viro-immunological status, comorbidities, and multidimensional geriatric assessment in FWLH. A complete case analysis was supplemented by multiple imputation using the mice package with the Predictive Mean Matching (PMM) method, and pooled estimates were derived from regression models, that included an interaction term for sex × birthplace.

**Results:**

We included 330 participants: 285 (86.5%) women, 15 (4.5%) FWLH and 30 (9%) FMLH. Comparing FWLH to IWLH, lower CD4+/CD8+ ratio (beta −0.38; 95% confidence interval (CI) −0.79, 0.03; p-value = 0.069) and percentage of CD4^+^ cell (beta −10; 95% CI -16, −4.1; p-value = 0.001) and higher weight (beta 11; 95% CI 3.4, 18; p-value = 0.004) and BMI (beta 3.8; 95% CI 0.57, 7.0; p-value = 0.021) were observed. Comparing FMLH to FWLH, we found lower prevalence of multimorbidity (IRR 0.60, 95% CI 0.37, 0.98, p-value = 0.039) and osteoporosis, though risk difference for osteoporosis was not significant. In the interaction model, FWLH had a lower percentage of CD4^+^ cells (β = −0.38; 95% CI: −0.73, −0.02; p = 0.036).

**Conclusion:**

FWLH in a geriatric cohort showed a profile of immune imbalance and higher weight, BMI, and multimorbidity; this may be possibly related to a worse metabolic profile and poorer access to care. However, there was no difference in virological response and antiretroviral therapies. Enhancing our understanding of older FWLH is crucial for promoting person-centered care a patient-centred care and healthy ageing in this population.

## Background

The global population aged ≥65 years is expected to rise from 10% in 2022 to 16% by 2050. In 2019, life expectancy at birth for women was 5.4 years longer than for men. The female survival advantage is present in all countries and regions, with differences ranging from 2.9 to 7 years (World Population Prospects, 2024). Therefore, due to longer life expectancy, the number of older women exceeds that of men. Globally, women accounted for 55.7% of people aged ≥65 years in 2022, although this percentage is expected to decline slightly to 54.5% by 2050. Notably, in Europe, about 16.2% of the foreign-born population is over 65 years of age (Migration d ata portal, 2023) and, in Italy, foreign-born residents account for 9% of the population (more than 5.3 million individuals) (Annuario statistico italiano, 2024). Following these demographic changes, the World Health Organization (WHO) has launched the United Nations Decade of Healthy Ageing (2021–2030) initiative to improve the lives of older people, their families, and their communities (WHO’s work on the UN Decade of Healthy Ageing, 2021).

These demographic changes are also evident in people living with HIV (PLWH), with an increasing prevalence of HIV in older populations. In the Dutch ATHENA Cohort (22,513 PLWH in 2023), 57% of the subjects were 50 years or older, of whom 49% were women, and 28% were aged 60 years or older. In 2002, only 20% of the cohort was aged over 50 (HIV Monitoring Report Stichting hiv monitoring, 2024). This increase is mainly due to improved antiretroviral therapy (ART), enabling long-term survival. Several European cohorts have also focused on this issue (Milic et al., 2019). Older PLWH represent a growing challenge for healthcare systems due to their complex clinical needs, including multimorbidity, polypharmacy, and functional decline. Effective engagement with healthcare services may help manage comorbidities and reduce long-term complications and costs. Although multiple non-communicable diseases are associated with higher healthcare utilization in the general population, it remains unclear whether this pattern holds true among older PLWH, who may face a disproportionately higher burden of chronic conditions (Yoo-Jeong et al., 2022). We previously explored sex-based differences in older PLWH, finding a worse virologic response despite a better immune reconstitution, as well as differences in terms of comorbidities and medications received, including ART (Focà et al., 2019).

Aging with HIV presents additional challenges related to late diagnosis and under-recognition, often due to low clinical suspicion among healthcare professionals and limited knowledge about age-specific risk factors. Contextual influences such as geographic location and healthcare access also impact diagnosis and treatment (Emlet, 2006; Hillman, 2007). Among PLWH in Europe, foreign-born individuals, mainly from regions with higher HIV prevalence (e.g., Sub-Saharan Africa or Eastern Europe) contribute to local epidemiological patterns. Migrant populations may be at greater risk for poor health outcomes due to structural and sociocultural barriers that limit their access to and navigation of healthcare systems in host countries. There is a critical lack of up-to-date epidemiological data on foreign-born populations, hindering the development of targeted public health strategies (MacKinnon et al., 2023). Aging foreign-born PLWH represent a vulnerable population that requires tailored medical, social, and policy interventions. To date, no studies have specifically addressed older foreign-born women living with HIV (FWLH) in Europe.

This study, therefore, aims to describe the characteristics of foreign-born women aged ≥65 years living with HIV (FWLH), and compare them with Italian women of the same age (IWLH) and foreign-born men (FMLH) within the GEriatric Patients living with HIV/AIDS cOhort (GEPPO). This is, to our knowledge, the first exploratory study of FWLH in a geriatric HIV cohort.

## Materials and methods

### Study population

This prospective, multicenter observational study used data from the GEriatric Patients living with HIV/AIDS cOhort (GEPPO), an Italian cohort including PLWH and community-dwelling controls aged ≥65 years, recruited across 12 HIV clinics and 8 geriatric care centers. Inclusion criteria were age ≥65 years, HIV infection, and continuous ART for at least 6 months. All participants are routinely offered geriatric assessments, but participation is voluntary and not all subjects consented to these additional evaluations. The cohort aims to characterize the health status and its progression in older PLWH.

We analyzed HIV-positive individuals enrolled between 2017 and 2024 grouped as FWLH, IWLH, and FMLH.

In Italy, foreign-born residents are categorized as Permanently Entitled Foreigners to Health Regional Assistance (PEF) and Temporarily Entitled Foreigners (TEF). Both groups have access to HIV care and ART.

Collected data included demographics, comorbidities, concomitant medications, and multidimensional geriatric assessments. Comorbidities assessed included chronic kidney disease, bone disease, dyslipidemia, type 2 diabetes, hypertension, and hypothyroidism. Geriatric assessments included the Pegboard test, the EuroQol 5-Dimension 5-Level (EQ-5D-5L) questionnaire (EQ-5D-5L User Guide, 2019), and evaluation of physical activity levels.

### Statistical analysis

Continuous variables were described using mean and standard deviation (SD), while categorical variables were described with absolute frequencies and percentages. The mice package (mice: Multivariate Imputation by Chained Equations) (Buuren and Groothuis-Oudshoorn, 2011) was used for multiple imputation of missing data, generating 15 imputed datasets with 15 iterations each, applying the Predictive Mean Matching (PMM) method. Analyses were performed in R (version 4.4.1). A complete case analysis was supplemented with this sensitivity analysis. For the imputation of CD4% and CD4/CD8 ratio, age, number of comorbidities, and polypharmacy status were included as predictors. Estimates were derived from pooled models built on the imputed datasets. The variables were chosen based on both theoretical relevance (intersectional disparities in health outcomes) and clinical plausibility. We used a beta regression for CD4^+^, a linear model for CD4+/CD8+ ratio, weight and body mass index (BMI), and a Poisson model for multimorbidity.

The models also included the interaction term (sex × birthplace). The model did not include additional covariates to avoid overfitting, given the limited sample size.

All statistical tests were performed assuming a two-sided 5% significance level. For each regression model, effect estimates are reported as point estimates together with 95% confidence intervals (95% CI) and corresponding p-values. Incidence rate ratios (IRR) and odds ratios (OR) are likewise presented with 95% CI. Pooled estimates from the multiple-imputation procedures are shown with 95% CI.

### Ethical disclosures

This study was conducted within the framework of the GEPPO cohort, utilizing data collected from enrolled participants and adhering to the cohort’s predefined scientific objectives (Guaraldi et al., 2018). The cohort obtained approval from the Research Ethics Board of each participating Centre. The Research Ethics Board of the coordinating centre was the Ethical Committee of Brescia (Italy) (NP 3135/2018). All the subjects signed a written informed consent. All data were fully anonymized before the statistical analysis was performed.

## Results

A total of 330 participants were included in the study: 285 (86.5%) women and 30 (9%) foreign-born men (FMLH). Among the women, 15 (4.5%) were foreign-born (FWLH).

Table 1 summarizes demographic and clinical characteristics. The mean age was comparable across groups. FWLH originated from South America (6 subjects, 40%), Africa (3 subjects, 20%), Eastern Europe (3 subjects, 20%), Western Europe (2 subjects, 13.3%), and Asia (1 subject, 6.7%).

**TABLE 1:** Demographic and clinical characteristics. IWLH, Italian women living with HIV; FWLH, foreign-born women living with HIV; FMLH, foreign-born men living with HIV. With respect to the multidimensional geriatric assessment, only a small proportion of participants completed at least one physical activity report. Continuous variables are presented as mean (SD).

Study population characteristics	IWLH, N = 285	FWLH, N = 15	FMLH, N = 30
Age	78.1 (±6.1)	76.5 (±4.4)	78.9 (±5.2)
Education years	9.1 (±5.7)	7.0 (NA)	NA
Weight	62 (±13)	73 (±16)	80 (±15)
BMI	25.0 (±5.5)	28.8 (±6·4)	26.3 (±5.0)
Annual weight gain	0.19 (±3.17)	−0.12 (±3.71)	−0.04 (±1.39)
N. Comorbidities	2.98 (±2.80)	2.47 (±1.85)	1.73 (±1.53)
Concomitant medications	2.32 (±2.56)	1.80 (±2.18)	1.73 (±1.53)
Pegboard test score	135 (±68)	123 (±58)	81 (NA)
Sedentary life	40/93 (43%)	3/5 (60%)	4/8 (50%)
Low physical activity	33/67 (49%)	2/4 (50%)	2/6 (33%)

HIV infection duration was similar in the three groups (17 years for IWLH, 15 years for FWLH, 18 years for FMLH). Over 95% had an undetectable viremia. FWLH showed a mean CD4^+^ cell count of 583 (±260) cells/mL, CD4^+^ 24% (±10), and a CD4+/CD8+ ratio of 0.65 (±0.35) (Table 2).

**TABLE 2:** Viro-immunological status. IWLH, Italian women living with HIV; FWLH, foreign-born women living with HIV; FMLH, foreign-born men living with HIV. Continuous variables are presented as mean (SD).

VIRO-IMMUNOLOGICAL status	IWLH, N = 285	FWLH, N = 15	FMLH, N = 30
HIV duration	17 (±8)	15 (±8)	18 (±8)
Unknown	21	0	0
HIV RNA <200cp/ml	153 (96%)	7 (100%)	18 (100%)
Unknown	125	8	12
CD4^+^ cell count	692 (±296)	583 (±260)	636 (±287)
Unknown	8	3	0
CD4^+^ %	36 (±11)	24 (±10)	28 (±9)
Unknown	7	3	0
CD4/CD8	1.08 (±0.67)	0.65 (±0.35)	0.66 (±0.44)
Unknown	28	3	4

**FIGURE 1:**
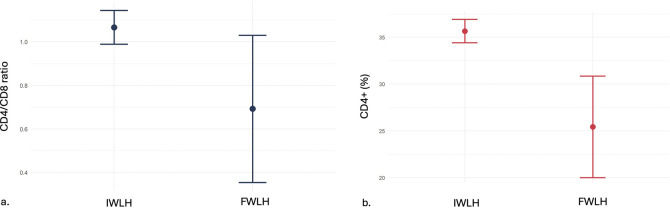
Immunological comparison between IWLH and FWLH. **(a)** Linear regression model for CD4+/CD8+ ratio **(b)** Linear regression model for CD4^+^ percentage. IWLH, Italian women living with HIV; FWLH, foreign-born women living with HIV. Mean and SD.

Comparing FWLH and IWLH with linear regression models, we did not evidence any difference in rate of HIV RNA undetectability, type of antiretroviral therapies, CD4^+^ cell count, prevalence of comorbidities (chronic kidney disease, bone disease, dyslipidaemia, type 2 diabetes mellitus, hypertension, and hypothyroidism), concomitant medications, annual weight gain, or access to multidimensional geriatric assessment. FWLH showed lower CD4+/CD8+ ratio (beta −0.38; 95% confidence interval (CI) −0.79, 0.03; p-value = 0.069) and CD4^+^ percentage (beta −10; 95% CI -16, −4.1; p-value = 0.001) (Figure 1). Moreover, IWLH had a lower mean weight (beta 11; 95% CI 3.4, 18; p-value = 0.004) and BMI (beta 3.8; 95% CI 0.57, 7.0; p-value = 0.021).

Comparing FWLH and FMLH, we did not evidence any difference in rate of HIV RNA undetectability, type of antiretroviral therapies, CD4^+^ cell count, CD4^+^ percentage, CD4+/CD8+ ratio, concomitant medications, weight, BMI, or access to multidimensional geriatric assessment.

**FIGURE 2:**
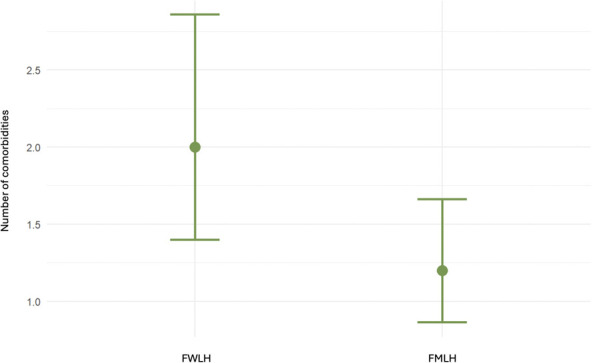
Comparison of the number of comorbidities between FWLH and FMLH. FWLH, foreign-born women living with HI, FMLH, foreign-born men living with HI. Mean and SD.

Compared with FMLH, FWLH showed higher multimorbidity risk (IRR 0.60, 95% CI 0.37, 0.98; p-value = 0.039) and osteoporosis prevalence (27% vs. 0%; p-value = 0.010), though risk difference for osteoporosis was not significant (OR 0.00; p-value >0.9) (Figure 2).

**FIGURE 3:**
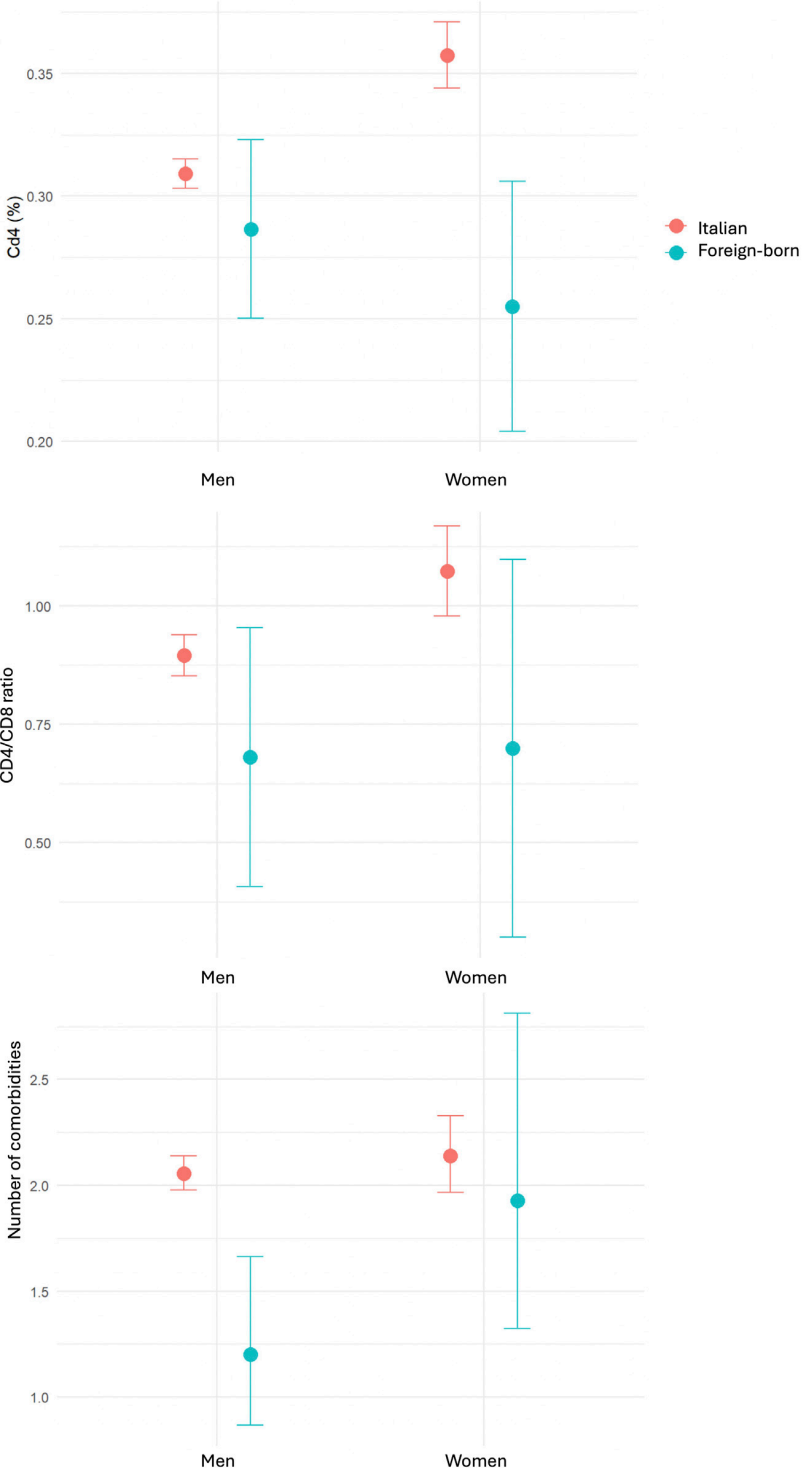
Interaction of factors “women” and “foreign-born” in the whole cohort. **(a)** CD4^+^ percentage, **(b)** CD4+/CD8+ ratio, **(c)** multimorbidity. Mean and SD.

Considering the interaction between “sex” and “birthplace”, FWLH had significantly lower CD4^+^ percentage (β = −0.38; 95% CI –0.73, −0.02; p = 0.036). The differences in multimorbidity (β = 1.54; 95% CI 0.93, 2.57; p = 0.093) and CD4+/CD8+ ratio (β = −0.16; 95% CI –0.69, 0.36; p = 0.50) were not statistically significant (Figure 3).

## Discussion

Older FWLH are highly understudied and face multiple layers of marginalization due to age, gender, immigration status, and HIV-related stigma. These intersecting vulnerabilities may contribute to disparities in treatment outcomes and access to care (Norcini-Pala et al., 1982; Conner et al., 2018).

To our knowledge, this is the first study to specifically describe foreign-born women aged ≥65 years living with HIV in Europe. Despite similar virological suppression, FWLH display poorer immunological profiles, with lower CD4^+^ percentages and CD4+/CD8+ ratios suggesting heightened immune activation. Furthermore, FWLH had a greater burden of multimorbidity compared to both IWLH and FMLH. The CD4/CD8 ratio is a well-established hallmark of immunosenescence and a predictor of morbidity and mortality, both in the general population and in PLWH (Serrano-Villar et al., 2014).

Given the exploratory nature of our study, we emphasize these immunological disparities and the observed multimorbidity burden as potential indicators of increased frailty, polypharmacy, and functional decline. These findings underscore the importance of developing targeted screening strategies and integrated care models tailored to the needs of aging, foreign-born women living with HIV.

While previous analyses of the GEPPO cohort reported a higher risk of age-related conditions among men, our results suggest this trend may reverse among foreign-born individuals, with FWLH exhibiting a greater prevalence of comorbidities such as osteoporosis (Focà et al., 2019). In addition to poorer immunometabolic profiles, these differences may also reflect structural barriers, including limited access to preventive services or delayed diagnoses (MacKinnon et al., 2023). Early detection of chronic conditions can slow progression of disease, reduce mortality, and reduce the burden on health systems (MacKinnon et al., 2023; Guaraldi et al., 2018; Serrano-Villar et al., 2014; Schouten et al., 2014; D’Arminio Monforte et al., 2019; De Francesco et al., 2019). Our results point to the urgent need for more proactive and equitable care models.

A better understanding of FWLH could support patient-centered care pathways and promote healthy aging.

Enhancing our understanding of FWLH could facilitate more person-centred care and promote healthier aging trajectories. For example, a study from rural South Africa found that PLWH with undetectable viral loads were more often diagnosed and treated for comorbidities such as hypertension and diabetes than HIV-negative peers, possibly due to the more frequent interaction with the healthcare services (D’Arminio Monforte et al., 2019). Long-term survivors of HIV may also exhibit greater health system literacy and resilience compared to individuals newly diagnosed with chronic conditions (Milic et al., 2019).

The heterogeneity within aging PLWH populations underscores the critical importance of adopting intersectional approaches in both research and clinical practice. Intersectionality recognizes that health outcomes are shaped not by a single factor (e.g., age, gender, HIV status), but by the dynamic and cumulative interplay of multiple identities and structural inequalities. Older foreign-born women living with HIV, for example, may simultaneously face ageism, sexism, racism, xenophobia, and HIV-related stigma, each compounding the others to produce unique vulnerabilities that affect access to care and health outcomes.

A U.S.-based study similarly emphasized the need to account for structural vulnerabilities in healthcare delivery for older women with HIV, highlighting the inadequacy of uniform approaches to care (Caiola et al., 2024).

Further efforts to integrate care for HIV and other chronic diseases may offer an avenue to improve outcomes for all. At an individual level, tailored education and empowerment programs should be developed to enhance health literacy, self-advocacy, and treatment adherence in FWLH. These interventions must be culturally and linguistically appropriate, addressing the unique needs of older women from diverse backgrounds.

At an interpersonal level, training healthcare professionals to recognize and respond to the complex identities and lived experiences of FWLH is essential. This includes anti-bias education, communication skills training, and the incorporation of patient narratives into care planning to build trust and therapeutic alliance.

At an institutional level, healthcare systems must implement policies that promote equitable access, such as the integration of HIV care with geriatric and social services, provision of professional interpreters, and outreach initiatives targeting underserved communities.

We recommend the integration of comprehensive geriatric assessments into routine HIV care to better address the complex needs of aging individuals. This should be accompanied by the greater use of multidisciplinary care teams, including geriatricians, infectious disease specialists, social workers, and mental health professionals, to provide comprehensive, coordinated care. We also propose culturally responsive services, including enhanced support structures for aging migrant populations with chronic conditions, to improve equity, care continuity, and long-term health outcomes. Data disaggregation by age, gender, and migration status should also be routine to inform inclusive policy and program design.

An intersectional framework not only improves clinical outcomes but also advances social justice, ensuring that healthcare delivery acknowledges and addresses the realities of those most at the margins. Future research and public health strategies must prioritize the voices and experiences of FWLH to inform interventions that are both evidence-based and equity-oriented.

Enhancing our understanding of FWLH is essential to fostering equitable, person-centered care. Our findings should inform the design of targeted clinical and policy interventions to support healthy aging in this neglected group. Further longitudinal studies are warranted to explore trajectories of aging in FWLH and assess the long-term impact of HIV, comorbidities, and social determinants of health in this population.

### Strengths and limitations

This study has several strengths. It draws on data from a large, multicenter cohort, enhancing its external validity and reflecting real-world clinical settings across Italy. Most notably, it provides a novel focus on a highly underrepresented and clinically relevant population: foreign-born women aged ≥65 years living with HIV. To our knowledge, this is the first study in a European context to explore the unique immunological and clinical profiles of this subgroup. By highlighting differences in immune activation and multimorbidity, this work contributes to a growing recognition of the need for tailored, patient-centered care approaches in older populations with HIV.

However, several limitations must be acknowledged. The small sample size limits the statistical power of our analyses and affects the precision and generalizability of our findings. We have therefore framed the study as a preliminary investigation, intended to generate hypotheses and stimulate further research, rather than draw definitive conclusions. Additionally, missing data affected several key variables. While we have addressed this in the manuscript, the lack of complete data, especially in the geriatric assessment component, reduces the robustness of some comparisons. Another important limitation is the absence of key demographic and socioeconomic information, such as duration of residence, immigration status, education, and healthcare access. These unmeasured confounders likely influence health outcomes and cannot be accounted for in our analysis. Furthermore, while all participants are routinely offered geriatric assessments, participation is voluntary, and not all subjects consented to these additional evaluations. This may have introduced selection bias into those components of the analysis. Finally, although a more detailed history of ART regimens and adherence patterns would have enriched the clinical context, we relied on currently available data, namely viral load and immunologic markers, which we believe still provide meaningful insight into the participants’ health status. Despite these limitations, we hope our findings will help raise awareness of this understudied subgroup and encourage more comprehensive and targeted research moving forward.

## Conclusion

Despite similar virological suppression, FWLH showed poorer immunological markers and greater multimorbidity, suggesting increased immune activation and vulnerability to aging-related complications. These findings highlight the intersectional challenges this group faces due to age, gender, migration, and HIV status. This study is the first to specifically describe older FWLH in a European context. While limited by small sample size and missing data, our study underscores the urgent need for integrated, culturally sensitive, and geriatric-informed HIV care. Future research should adopt intersectional approaches to guide equitable health interventions and support healthy aging in this underserved population.

## Data Availability

The study protocol and de-identified dataset are available from the corresponding author on reasonable request. Requests to access the datasets should be directed to emanuele.foca@unibs.it.

## Group member of The GEPPO Study group

Stefania Arsuffi, Domenico Azzolino, Marta Baroni, Giuseppe Bellelli, Luca Bonaffini, Paolo Bonfanti, Andrea Calcagno, Stefano Calza, Annamaria Cattelan, Benedetto Maurizio Celesia, Alessandra Coin, Giuseppe De Socio, Giovanni Di Perri, Micol Ferrara, Benedetta Fioretti, Emanuele Focà, Giovanni Guaraldi, Francesca Italiani, Alessandro Lazzaro, Tiziano Lucchi, Maria Grazia Maddalone, Giordano Madeddu, Alessandra Marengoni, Claudio Mastroianni, Jovana Milić, Chiara Mussi , Silvia Nozza, Giancarlo Orofino, Lavinia Patetta, Andrea Piazzoli, Stefania Piconi, Paola Pignata, Silvia Pontiggia, Agostino Riva, Anna Spolti.
