# Correction: Health profiles of foreign-born elderly women with HIV in Italy

**DOI:** 10.3389/fragi.2025.1727256

**Published:** 2025-11-07

**Authors:** Stefania Arsuffi, Eugenia Quiros-Roldan, Fabio Riccardo Colombo, Benedetta Fioretti, Caterina Candela, Benedetto Maurizio Celesia, Micol Ferrara, Jovana Milic, Giuseppe Vittorio De Socio, Giordano Madeddu, Anna Maria Cattelan, Stefania Piconi, Paolo Bonfanti, Agostino Riva, Giovanni Guaraldi, Stefano Calza, Andrea Calcagno, Emanuele Focà, GEPPO Cohort

**Affiliations:** 1 Department of Clinical and Experimental Sciences, Unit of Infectious Diseases, University of Brescia and Spedali Civili Hospital, Brescia, Italy; 2 Unit of Biostatistics and Bioinformatics, Department of Molecular and Translational Medicine, University of Brescia, Brescia, Italy; 3 Clinic of Infectious Diseases, San Raffaele Scientific Institute, Milano, Italy; 4 Division of Infectious Diseases, ARNAS Garibaldi, Catania, Italy; 5 Unit of Infectious Diseases, Department of Medical Sciences, University of Torino, Torino, Italy; 6 Department of Surgical, Medical, Dental and Morphological Sciences, University of Modena and Reggio Emilia, Modena, Italy; 7 Department of Medicine, Clinic of Infectious Diseases, University of Perugia, Perugia, Italy; 8 Unit of Infectious Diseases, Department of Medicine, Surgery and Pharmacy, University of Sassari, Sassari, Italy; 9 Infectious and Tropical Diseases Unit, Padua University Hospital, Padova, Italy; 10 Infectious Diseases Unit, Alessandro Manzoni Hospital, Lecco, Italy; 11 Fondazione IRCCS San Gerardo dei Tintori, Monza - University of Milano-Bicocca, Milan, Italy; 12 Third Division of Infectious Diseases, University of Milan, Ospedale L. Sacco, Milan and University of Milan, Milan, Italy

**Keywords:** HIV, women, foreign-born, aging, healthy longevity

**Affiliation 8** was erroneously given as “Unit of Immune Deficiency, Department of Medicine, Surgery and Pharmacy, University of Sassari, Sassari, Italy”. **Affiliation 8** has been corrected to “Unit of Infectious Diseases, Department of Medicine, Surgery and Pharmacy, University of Sassari, Sassari, Italy”.

Author **Giordano Madeddu** was erroneously spelled as Giordano Maddeddu

The original article has been updated.

